# Facile one‐step synthesis of efficient hybridized local and charge transfer emitter for ultraviolet electroluminescence with low roll‐off

**DOI:** 10.1002/smo2.70090

**Published:** 2026-08-22

**Authors:** Shengnan Wang, Jixiang Wang, Danyu Xie, Ling Peng, Yuchao Liu, Junjie Wang, Shian Ying, Dongge Ma, Shouke Yan

**Affiliations:** ^1^ Key Laboratory of Rubber‐Plastics, State Key Laboratory of Advanced Optical Polymer and Manufacturing Technology, Ministry of Education Qingdao University of Science and Technology Qingdao China; ^2^ State Key Laboratory of Luminescent Materials and Devices, Guangdong Provincial Key Laboratory of Luminescence from Molecular Aggregates, Center for Aggregation‐Induced Emission South China University of Technology Guangzhou China; ^3^ College of Chemistry and Chemical Engineering Heze University Heze China; ^4^ State Key Laboratory of Chemical Resource Engineering, College of Materials Science and Engineering Beijing University of Chemical Technology Beijing China

**Keywords:** efficiency roll‐off, hybridized local and charge transfer, non‐covalent interaction, organic light‐emitting diode, ultraviolet emitter

## Abstract

Ultraviolet organic light‐emitting diodes (UV‐OLEDs) have attracted increasing attention in the applications of phototherapy, high‐density information storage, sterilization, photocuring and so on, yet the development of efficient short‐wavelength emitters remains challenging due to the irreconcilable contradiction between fluorescence efficiency and short molecular conjugation length. Here, we design and synthesize a simple yet efficient donor–acceptor–donor (D–A–D) type UV emitter D3CZ4F featuring a hybridized local and charge transfer state, via a facile one‐step coupling reaction. The multiple intramolecular non‐covalent C–H⋯F locking interactions enhance molecular rigidity as well as promote the planarization of the excited state geometry, leading to a large oscillator strength of 1.3989 and a high solid‐state photoluminescence quantum yield of 86%. Consequently, the doped device with D3CZ4F as an emitter not only emits high color‐purity UV emission at 388 nm with color coordinates of (0.165, 0.025), but also furnishes a maximum external quantum efficiency (EQE) of up to 8.97% and a small roll‐off with the EQE of 8.15% at 500 cd m^−2^. Moreover, the non‐doped device achieved a surprised EQE of 7.33% with an emission peak at 390 nm and color coordinates of (0.164, 0.027), respectively.

## Graphical abstract

An effective hybridized local and charge‐transfer emitter D3CZ4F featuring multiple C–H⋯F locking interactions was developed, achieving an outstanding EQE of 8.97%, a small roll‐off, an emission peak at 388 nm, and color coordinates of (0.165, 0.025), respectively.

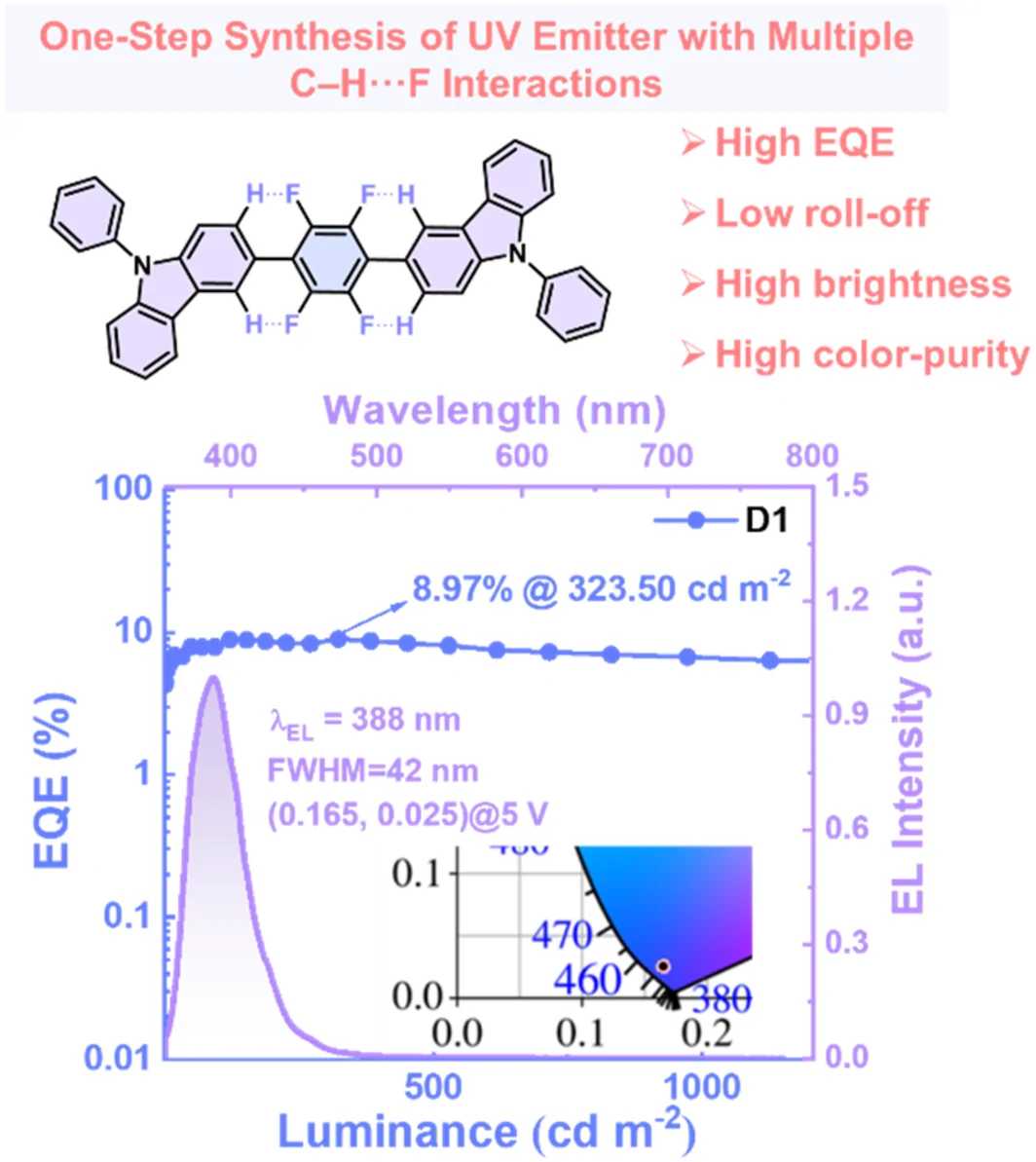

## 1 INTRODUCTION

Ultraviolet (UV) luminescent materials have garnered significant research attention owing to their promising applications in medical phototherapy, environmental purification, excitation light sources, and high‐density data storage.[^1^, ^2^, ^3^, ^4^, ^5^] Compared to conventional mercury lamps and inorganic light‐emitting diodes (LEDs), organic light‐emitting diodes (OLEDs) offer distinctive advantages such as self‐emission, low power consumption, lightweight flexibility, and designable molecular structures, making them ideal candidates for next‐generation UV light sources.[^6^, ^7^, ^8^, ^9^, ^10^, ^11^] However, in contrast to their red, green, and blue counterparts, the variety and quantity of high‐performance UV emitters remain scarce, and electroluminescence (EL) efficiencies of ultraviolet organic light‐emitting diodes (UV‐OLEDs) still lag. As delineated by the expression for external quantum efficiency (EQE):

(1) $$\text{EQE}=\gamma \times \eta_{\text{out}}\times \eta_{r}\times \Phi_{\text{PLQY}},$$

where *γ* is the charge recombination efficiency (ideally 100%). *η*_out_ is the light out‐coupling efficiency (20%–30% for bottom‐emitting OLEDs), *η*_r_ is the radiative exciton probability, which characterizes the efficiency of excitons, Φ_PLQY_ is the photoluminescence quantum yield (PLQY) of the emitter, respectively. The construction of UV emitters with high PLQY is one of the critical factors for achieving high‐performance UV‐OLEDs with a high EQE. However, high PLQY typically requires extended π‐conjugation lengths to enhance electron delocalization, whereas short‐wavelength UV emission necessitates short π‐conjugation lengths in emitters. This inherent contradiction poses significant challenges in designing UV emitters that simultaneously achieve high PLQY and short‐wavelength UV emission. Recent efforts to enhance PLQY have leveraged a diverse array of molecular design strategies, including using bulky substituents to suppress π–π stacking, designing planarized intramolecular charge transfer state emitters based on donor′–acceptor–donor (D′–D–A) architecture, constructing rigid coplanar emitter with medium‐range CT state, developing aggregation‐induced emission enhancement (AIEE)‐active emitters, and utilizing “crossed long‐short axis” molecular strategy.[^12^, ^13^, ^14^, ^15^, ^16^] Although the aforementioned strategies have achieved partial enhancement in PLQY, reported UV emitters rarely achieve PLQY values exceeding 80% in the film state.

**FIGURE 1:**
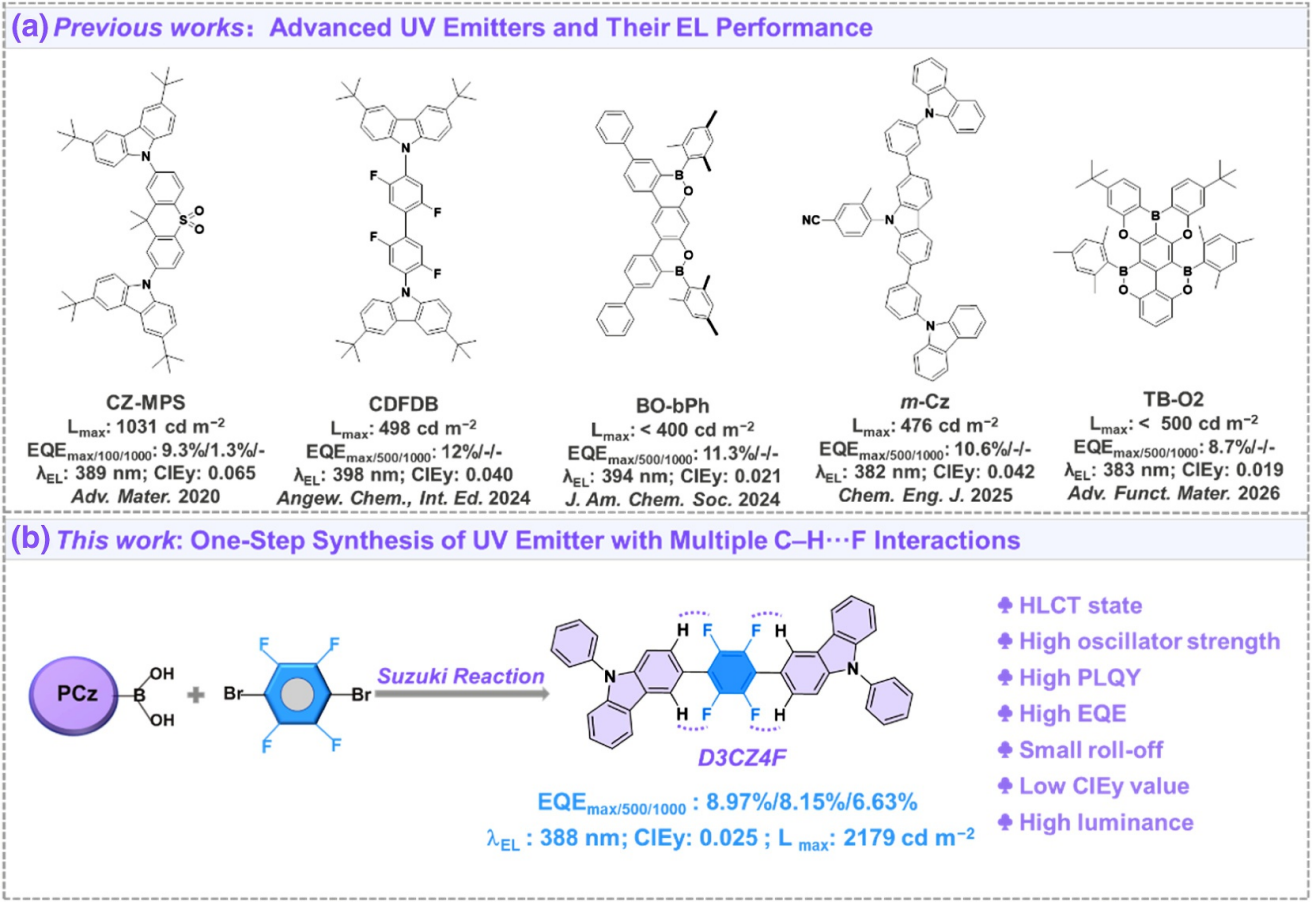
(a) Reported UV emitters with high EL performance. (b) Schematic of the molecular design principle of D3CZ4F. EL, electroluminescence; UV, ultraviolet.

The effective utilization of 75% of electrically generated triplet excitons constitutes another critical factor for achieving high EQE in pure organic EL materials. To date, various triplet‐harvesting material systems have been developed, including triplet‐triplet annihilation (TTA), thermally activated delayed fluorescence (TADF), room‐temperature phosphorescence, and hybridized local and charge‐transfer (HLCT) materials.[^17^, ^18^, ^19^, ^20^, ^21^, ^22^, ^23^, ^24^, ^25^] In particular, HLCT materials enabling triplet exciton harvesting via high‐lying reverse intersystem crossing (hRISC) demonstrate exceptional promise in UV‐OLEDs.[^4^, ^12^, ^23^, ^26^, ^27^, ^28^, ^29^, ^30^] In this molecule system, localized excited (LE) state component effectively preserves higher exciton energy and fast radiative decay process, achieving high PLQYs, while CT state component facilitate efficient hRISC from high‐energy triplet states for utilizing dark‐state triplet excitons.[^13^, ^31^, ^32^, ^33^, ^34^, ^35^, ^36^, ^37^] There are a few reported high‐efficiency OLEDs that have achieved a maximum EQE of over 10% and stable UV emission below 400 nm. For example, Zhang et al. reported a long‐short axis UV emitter 2BuCz‐CNCz, exhibiting an EL peak at 396 nm alongside a maximum EQE as 10.79%.^[38]^ Zhang et al. developed a linear D–A–D type emitter 9,9'‐(2,2',5,5'‐tetrafluoro‐[1,1'‐biphenyl]‐4,4'‐diyl)bis(3,6‐di‐tert‐butyl‐9*H*‐carbazole) (CDFDB), achieving UV emission at 398 nm with an EQE of 12.0%.^[6]^ Li et al. harnessed the embedding of bis‐B–O units into non‐linear polycyclic frameworks to develop BO‐bPh, which elicits EL peaking at 394 nm with a maximum EQE of 11.3%.^[36]^ Zhan et al. engineered the rigid, planar double B–O–N polycyclic architecture of emitter BO–N, which, as the doped device, yields an EL peak at 399 nm and a maximum EQE of 18.6%.^[37]^ Although the above‐mentioned UV‐OLEDs have achieved certain breakthroughs in maximum EQE, their emission peaks are predominantly concentrated in the 390–400 nm range, and the efficiency roll‐off at high luminance levels is particularly severe, with EQE dropping below 5% at 500 cd m^−2^ (Figure 1a).

Hence, we present a straightforward yet highly efficient donor‐acceptor‐donor (D–A–D) type HLCT UV emitter, 3,3′‐(perfluoro‐1,4‐phenylene)bis(9‐phenyl‐9*H*‐carbazole) (D3CZ4F), which was synthesized via a one‐step Suzuki coupling reaction (Figure 1b). The enhanced intramolecular non‐covalent C–H⋯F interactions promote more planarized conformation from the ground state to the lowest singlet excited state, enhancing the structural rigidity and π‐electron delocalization. This endows D3CZ4F with a high oscillator strength and fluorescence efficiency while maintaining short‐wavelength UV emission. Accordingly, D3CZ4F exhibits a UV emission with peak and PLQY of 388 nm and 98% in solution, and 379 nm and 86% in doped film, respectively. The doped device achieved a maximum EQE of 8.97% with an EL peak at 388 nm, a full‐width at half‐maximum (FWHM) of 42 nm, and Commission Internationale de I’Eclairage (CIE) coordinates of (0.165, 0.025). The EQE remained at 8.15% and 6.63% at 500 and 1000 cd m^−2^. Owing to the high horizontal dipole orientation of the D3CZ4F neat film, the D3CZ4F‐based non‐doped OLED achieved a maximum EQE of 7.33%, an EL peak at 390 nm, and CIE coordinates of (0.164, 0.027), respectively.

## 2 RESULTS AND DISCUSSION

### 2.1 Synthesis and characterization

Supporting Information S1: Scheme S1 outlines the straightforward synthetic route to D3CZ4F, which was efficiently prepared via a one‐step palladium‐mediated Suzuki coupling reaction using low‐priced 9‐phenylcarbazole‐3‐boronic acid and 1,4‐dibromotetrafluorobenzene raw materials. After purification by column chromatography and vacuum gradient sublimation, the molecular structure was unambiguously confirmed by ^1^H NMR, ^13^C NMR, and high‐resolution mass spectrometry (See Supporting Information S1: Figure S11). Thermogravimetric analysis measurements revealed that D3CZ4F exhibited a decomposition temperature (*T*_d_, 5% weight loss) of 429°C, while a distinct endothermic signal was observed during the second‐heating curve of differential scanning calorimetry measurements, corresponding to the melting point of 296°C (Supporting Information S1: Figure S1). According to the onset of the oxidation wave from cyclic voltammetry (CV) measurement (Supporting Information S1: Figure S2), the highest occupied molecular orbital (HOMO) energy level of D3CZ4F was estimated to be −5.27 eV. The lowest unoccupied molecular orbital (LUMO) energy level was calculated to be −1.80 eV by the energy difference between the HOMO and optical bandgap (*E*_g_).

### 2.2 Theoretical calculations

**FIGURE 2:**
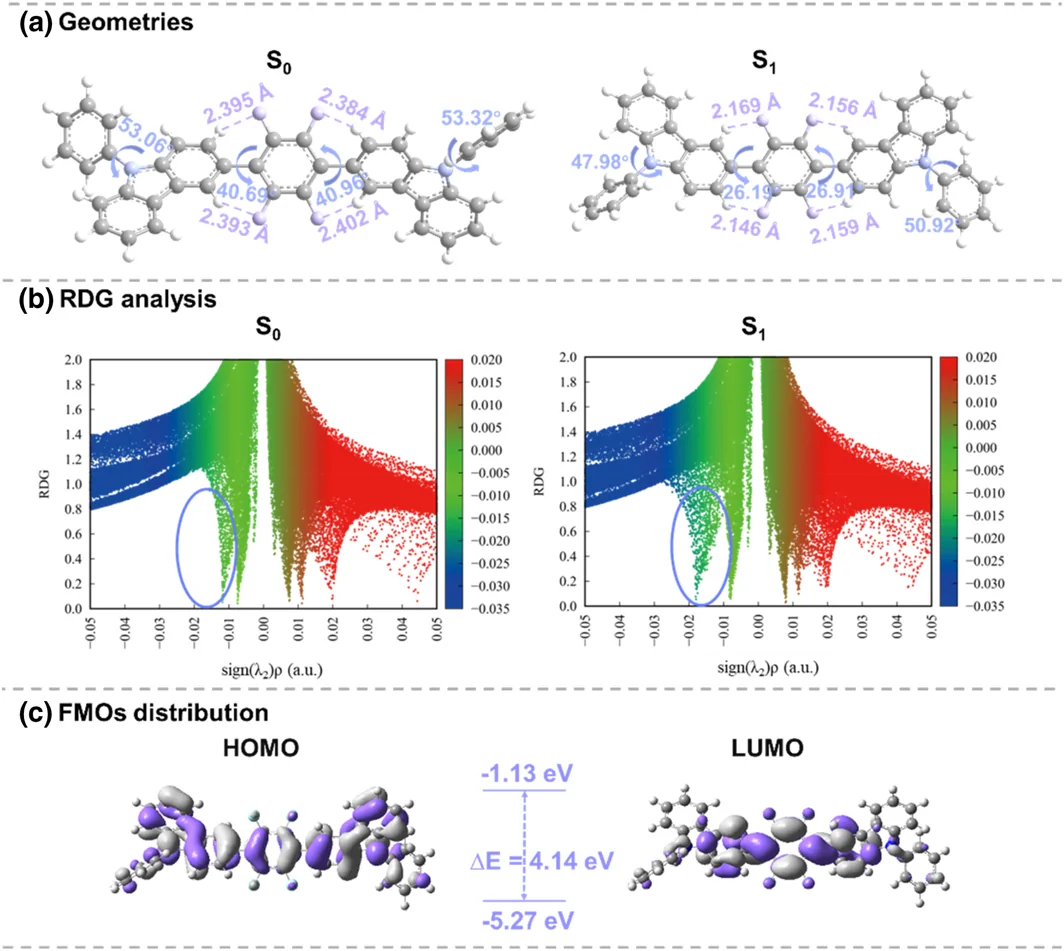
(a) The optimized *S*_0_ and *S*_1_ geometries of D3CZ4F. (b) RDG Scattering diagrams based on optimized *S*_0_ and *S*_1_ geometries of D3CZ4F. (c) FMO distributions and calculated energy levels of D3CZ4F. FMO, Frontier molecular orbital; RDG, reduced density gradient.

To elucidate the geometric structure and electronic properties of D3CZ4F, density functional theory (DFT) and time‐dependent DFT (TD‐DFT) calculations were carried out at the B3LYP/6‐31G(d, p) level. Natural transition orbital (NTO) analyses were performed using Multiwfn 3.8.^[39]^ In the ground state (*S*_0_), D3CZ4F adopts a moderately twisted conformation, with dihedral angles of 40.69° and 40.96° between the tetra‐fluorobenzene and carbazole units (Figure 2a). The calculated C–H⋯F distances of 2.384–2.402 Å suggest the presence of intramolecular C–H⋯F hydrogen‐bonding interactions.^[6]^ Upon photoexcitation, the optimized *S*_1_ geometry shows a notable planarization tendency where the dihedral angles between tetrafluorobenzene and carbazole reduce to 26.19° and 26.91°, and the C–H⋯F distances shorten to 2.146–2.169 Å (Figure 2a), which is conducive to rigidifying the molecular framework, promoting electron delocalization and reducing intramolecular vibrational modes, thereby facilitating high fluorescence efficiency for D3CZ4F. Reduced density gradient analysis of the optimized *S*_0_ further confirms non‐covalent C–H⋯F interactions are enhanced in the *S*_1_ geometry where the green region shows the presence of obvious attractive interactions (Figure 2b). As illustrated in Figure 2c, the HOMO is delocalized across the entire π system, whereas the LUMO is primarily localized on the tetrafluorobenzene acceptor and its directly linked benzene rings, resulting in substantial orbital overlap in tetrafluorobenzene and carbazole.

**FIGURE 3:**
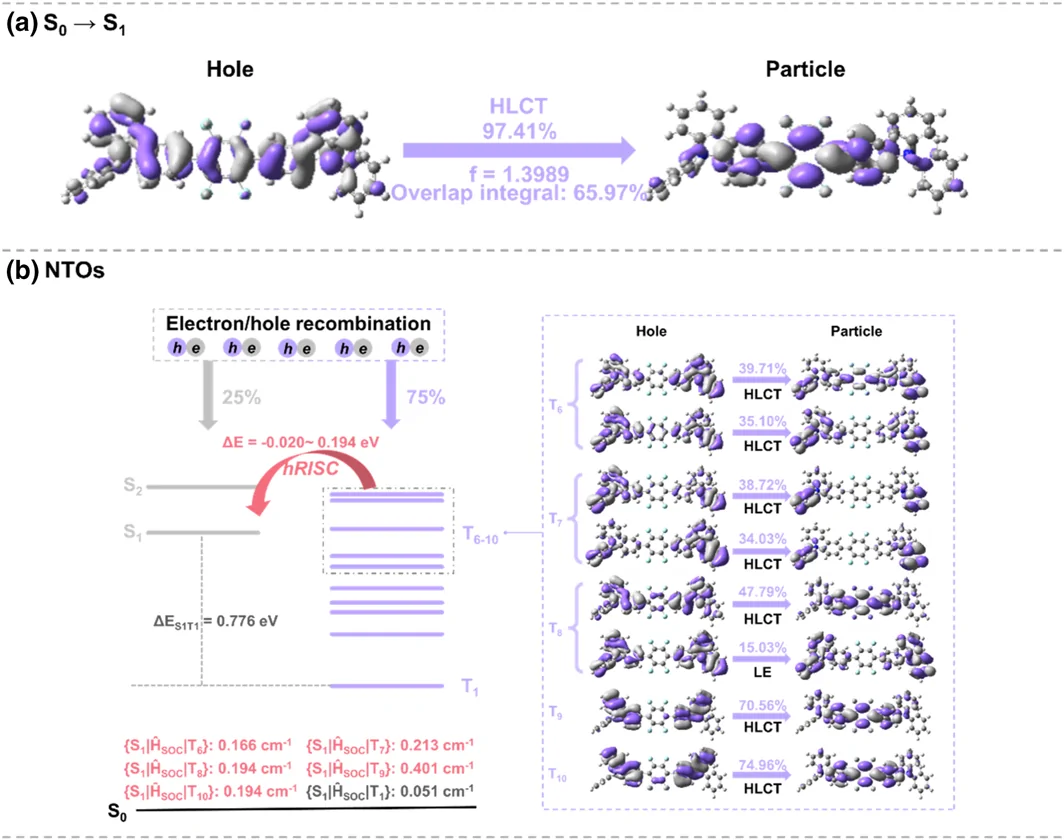
(a) The NTO analysis for *S*_0_ → *S*_1_ of D3CZ4F. (b) Proposed mechanism for the multi hRISC channels, NTO distributions, energy level diagram of excited states, and the SOC matrix elements between *S*_1_ and *T*_6_–*T*_10_ states. hRISC, high‐lying reverse intersystem crossing; NTO, natural transition orbital; SOC, spin–orbit coupling.

In the *S*_1_ state, there exists the remarkable orbital overlap and some separation between hole and particle distributions (Figure 3), illustrating that D3CZ4F exhibits the typical HLCT excited state characteristic where the proportions of LE and CT components are 65.97% and 34.03%, respectively. Such a weak CT component endows D3CZ4F with a high oscillator strength (*f* = 1.3989), which is favorable to obtain high PLQY. As shown in Figure 3 and Supporting Information S1: Table S1, D3CZ4F exhibits a large energy gap ($\Delta E_{S_{1}T_{1}}$: 0.776 eV) and a small spin–orbit coupling (SOC) matrix element (0.051 cm^−1^) between *S*_1_ and *T*_1_, suggesting that the TADF mechanism involving the RISC process from *T*_1_ to *S*_1_ can be effectively ruled out. Notably, as evidenced by the hole and particle distributions, the high‐energy triplet states *T*_6_–*T*_10_ predominantly exhibit HLCT state character, which have minimal energy gaps (ranging from −0.020 to 0.194 eV) with *S*_1_ state. In addition, the SOC matrix elements between *S*_1_ and these triplets are large, reaching 0.166, 0.213, 0.194, 0.401, and 0.194 cm^−1^, respectively. These conditions collectively demonstrate the potential for facilitating the hRISC processes in the hot exciton channels from *T*_6_–*T*_10_ to *S*_1_.

### 2.3 Photophysical properties

**FIGURE 4:**
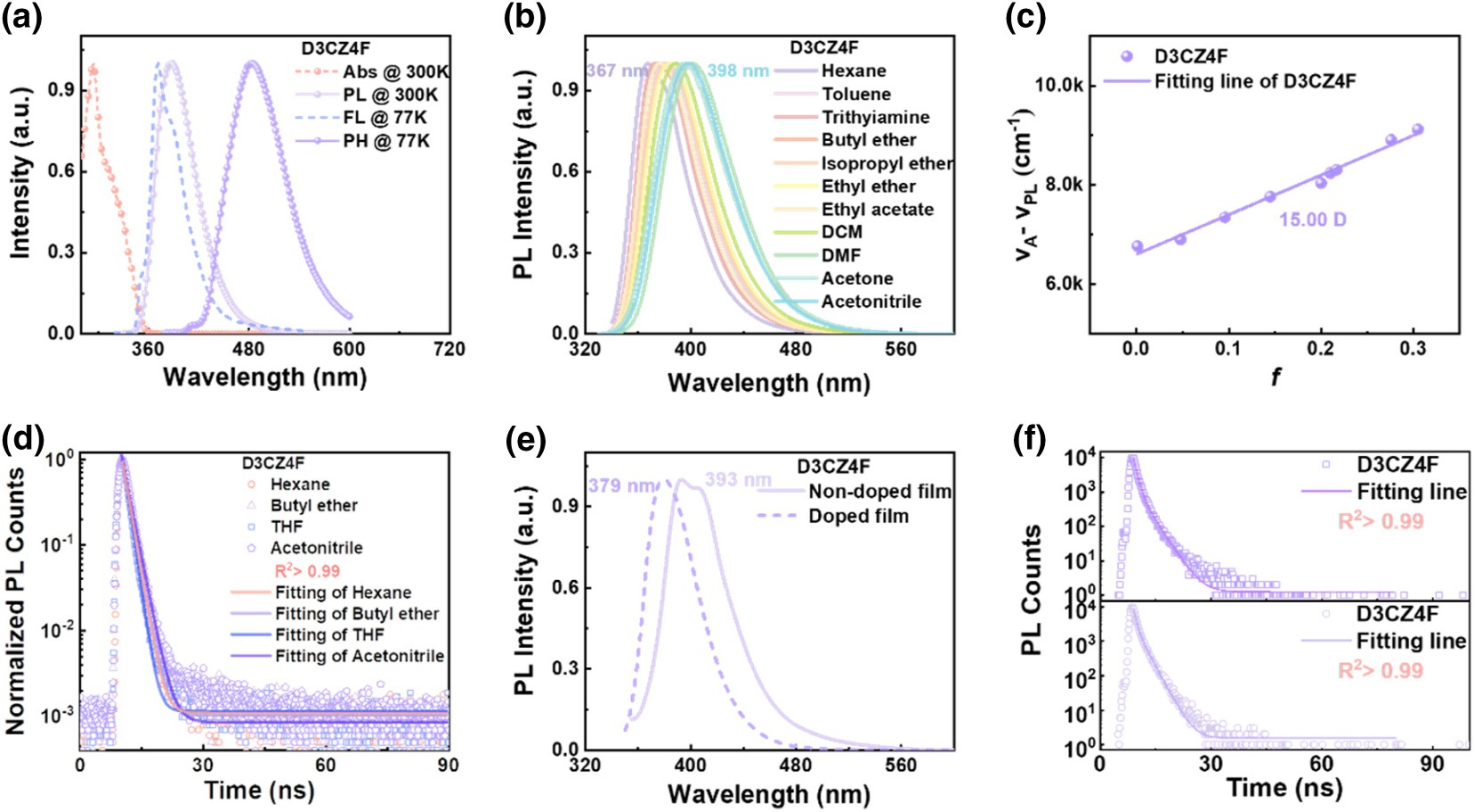
(a) UV–vis absorption and PL spectra at room temperature, and low‐temperature fluorescent and phosphorescent spectra at 77 K of D3CZ4F in THF solution (1.0 × 10^−5^ M). (b) Solvatochromic PL spectra. (c) Solvatochromic Lippert–Mataga model of D3CZ4F. (d) Transient PL decay spectra of D3CZ4F in different solvents (1.0 × 10^−5^ M). (e) PL spectra of D3CZ4F in non‐doped and doped films. (f) Transient PL decay spectra of D3CZ4F in non‐doped and doped films in vacuum. PL, photoluminescence; THF, tetrahydrofuran; UV, ultraviolet.

As illustrated in Figure 4a, the UV‐Visible (UV‐vis) absorption and room‐temperature photoluminescence (PL) spectra in dilute tetrahydrofuran (THF, 1.0 × 10^−5^ M) of D3CZ4F were examined. Absorption bands centered at 294 and ∼320 nm are assigned to π–π* and n–π* transitions of the phenyl‐carbazole moiety, respectively.^[40]^ The *E*_g_ deduced from the absorption onset can be determined to be 3.47 eV. The PL spectrum shows a UV emission peak at 388 nm with an exceptionally high PLQY of 98%. As shown in Supporting Information S1: Figure S3, compared to the PL spectrum in air atmosphere, the PL intensity in nitrogen atmosphere is significantly enhanced, representing that the triplet excitons participate in the radiative decay process. Low‐temperature fluorescence and phosphorescence spectra allow the determination of the *S*_1_ and *T*_1_ energy levels as 3.33 and 2.56 eV, respectively, yielding a large $\Delta E_{S_{1}T_{1}}$ of 0.77 eV, which implies that the TADF mechanism is difficult to realize. To probe the nature of the excited state, solvatochromic studies were conducted (Figure 4b and Supporting Information S1: Figure S4). With increasing solvent polarity, there is no significant red shift in the absorption peak, but the fluorescence peak redshifts from 367 nm with weak vibronic structures in n‐hexane solution to 398 nm with featureless emission profiles in acetonitrile solution, confirming a substantial CT component in the excited state.[^41^, ^42^] Applying the Lippert‐Mataga equation to the experimental data (Figure 4c; Supporting Information S1: Table S2) reveals a single linear correlation across the entire polarity range, giving an excited state dipole moment (*μ*_e_) of 15.00 D, which is smaller than that of typical CT material 4‐(*N,N*‐dimethylamino)benzonitrile (≈23 D),[^43^, ^44^] illustrating a quasi‐equivalent hybridization between LE and CT configurations.[^31^, ^45^, ^46^, ^47^, ^48^] Moreover, fluorescence decay profiles in both low and high polarity solvents display single‐exponential nanosecond‐level life‐times without any delayed components (Figure 4d), affirming that emission originates from a single HLCT state rather than mixed states between LE and CT components.

As shown in Figure 4e, D3CZ4F still maintains desirable UV emission with a maximum peak at 393 nm in a neat film, showing a slight bathochromic‐shift of 5 nm compared to that in THF, which may be ascribed to the suppressed intermolecular aggregation effect induced by the large torsion angles within phenylcarbazole segments. In the doped film with a concentration of 5 wt%, D3CZ4F exhibits a blue shifted UV emission peak at 379 nm with a FWHM of 47 nm, accompanied by a considerable PLQY of 86%. Transient PL decays of both neat and doped films under vacuum were performed in the nanosecond‐ and microsecond‐range detection window (Figure 4f, Supporting Information S1: Figure S5). There shows no microsecond scale delayed fluorescence; instead, they display biexponential nanosecond decays with the prompt and delay lifetimes of 1.10 and 4.48 ns for neat film, and 1.07 and 5.23 ns for doped film), respectively. The former can be attributed to direct singlet emission while the letter is attributed to the radiative transition of singlet excitons formed from the hRISC process. The radiative decay rates (*k*_r_) were calculated as 1.85 × 10^8^ and 6.96 × 10^8^ s^−1^, and the corresponding hRISC rates (*k*_hRISC_) reach 2.7 × 10^7^ and 2.2 × 10^7^ s^−1^, for neat and doped films, respectively (Table 1, Supporting Information S1: Table S3). These rapid *k*_r_ and *k*_hRISC_ can effectively reduce the accumulation of excitons in the light‐emitting layer, thereby reducing the efficiency roll off in the OLEDs.[^49^, ^50^, ^51^] Notably, compared to 3,3′‐(2,5‐difluoro‐1,4‐phenylene)bis(9‐phenyl‐9*H*‐carbazole) (D3CZ2F) lacking the two fluorine atoms,^[52]^ the *k*_r_ and *k*_hRISC_ for the D3CZ4F doped film were significantly improved, leading to a higher PLQY, which further verified the effect of multiple C‐H···F interactions in D3CZ4F.

**TABLE 1:** Key photophysical and electrochemical properties of D3CZ4F.

Compound	Solution^a^			Neat^b^/doped^c^ film			HOMO/LUMO/*E*_g_ ^d^ [eV]	$E_{S_{1}}$/$E_{T_{1}}$/$\Delta E_{S_{1}T_{1}}$ ^e^ [eV]
	*λ*_Abs_ [nm]	*λ*_Fluo_ [nm]	PLQY [%]	*λ*_Fluo_ [nm]	PLQY [%]	*τ* [ns]		
D3CZ4F	294	388	98	393/379	25/86	1.73/1.63	−5.27/−1.80/3.47	3.33/2.56/0.77

Abbreviations: HOMO, highest occupied molecular orbital; LUMO, lowest unoccupied molecular orbital; PLQY, photoluminescence quantum yield; THF, tetrahydrofuran.

^a^ Measured in a dilute THF solution (1.0 × 10^−5^ M) at 300 K.

^b^ Measured in neat film.

^c^ Measured in doped film.

^d^ HOMO energy level obtained from CV measurement. LUMO energy level calculated by *E*_LUMO_ = *E*_HOMO_ + *E*_g_.

^e^ Estimated by the peaks of the low‐temperature fluorescence and phosphorescence spectra at 77 K, $\Delta E_{S_{1}T_{1}}$ = $E_{S_{1}}$ − $E_{T_{1}}$.

### 2.4 EL performance

**FIGURE 5:**
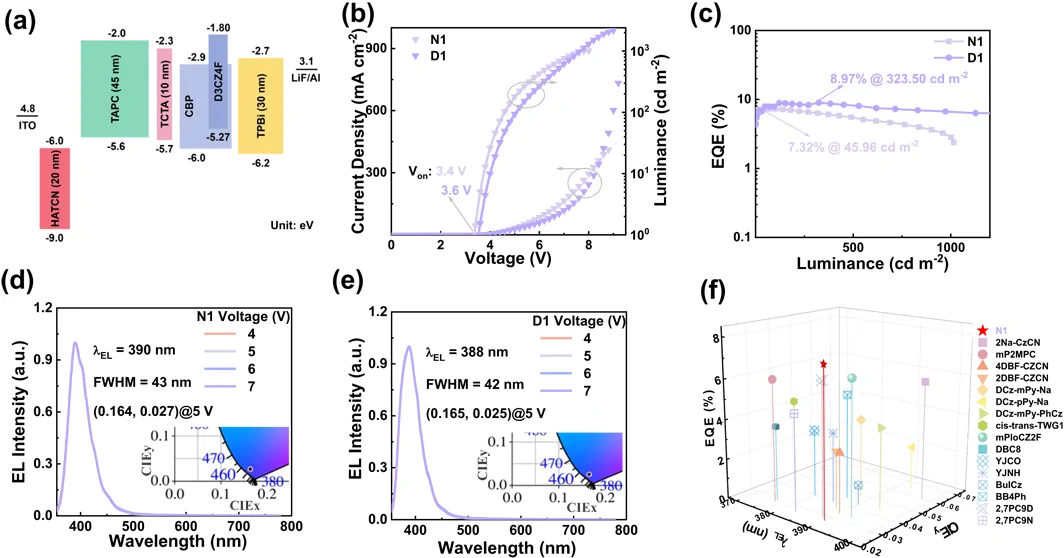
(a) Energy level alignment of the materials used in the OLEDs. (b) Current density voltage‒luminance characteristics of the devices N1 and D1. (c) EQE‐luminance characteristics of the devices N1 and D1. (d) EL spectra of the device N1 at different voltages (Insert: CIE coordinate at 5 V). (e) EL spectra of the device D1 at different voltages (Insert: CIE coordinate at 5 V). (f) Summary of EQE_max_ and CIE_*y*_ the representative reported non‐doped UV‐OLEDs with an EL peak ≤ 400 nm. CIE, Commission Internationale de I’Eclairage; EL, electroluminescence; EQE, external quantum efficiency; UV‐OLEDs, ultraviolet organic light‐emitting diodes.

Considering the high thermal stability, excellent PLQY, large *k*_r_, and efficient hRISC of D3CZ4F, we fabricated non‐doped and doped bottom‐emitting devices (N1 and D1) to characterize its EL characteristics with the following architecture: indium tin oxide/1,4,5,8,9,11‐hexaazatriphenylenehexacarbonitrile (ITO/HATCN) (20 nm)/di‐(4‐(*N,N*‐dimethyl‐4‐amino)‐phenyl)cyclohexane (TAPC) (45 nm)/tri(4‐carbazolyl‐9‐ylphenyl)amine (TCTA) (10 nm)/emissive layer (20 nm)/1,3,5‐tris(1‐phenyl‐1*H*‐benzimidazol‐2‐yl)benzene (TPBi) (30 nm)/lithium fluoride (LiF) (1 nm)/Al (100 nm). Among them, ITO and aluminum (Al) served as anode and cathode; HATCN and LiF were used as hole and electron injection layers; TAPC and TPBi were functioned as hole and electron transport layers; TCTA acted as an exciton blocking layer. The emissive layer of the doped device D1 comprises non‐polar 4,4'‐bis(9‐carbazolyl)‐1,1'‐biphenyl (CBP), as the host matrix with a low doping concentration of 5 wt%, which can further alleviate the exciton quenching induced by intermolecular host‐guest dipole‐dipole interactions. The corresponding energy‐level alignment and layer‐by‐layer layout are depicted in Figure 5 and Supporting Information S1: Figure S6. The EL performances are shown in Figure 5, Supporting Information S1: Figure S7 and summarized in Table 2.

**TABLE 2:** Summary of the EL performance of non‐doped device N1 and doped device D1.

Devices	*V*_on_ [V]	CE_max_ [cd A^−1^]	PE_max_ [lm W^−1^]	EQE_max/500/1000_ [%]	*λ*_EL_ [nm]	FWHM [nm]	CIE (*x*, *y*)	*R*_max_ [mW cm^−2^]	*R*_UV_ ^a^ [mW cm^−2^]
N1	3.4	0.55	0.51	7.33/5.40/2.82	390	43	(0.164, 0.027)	58.47	34.50
D1	3.6	0.54	0.24	8.97/8.15/6.63	388	42	(0.165, 0.025)	150.81	96.52

Abbreviations: *λ*_EL_, EL peak at 5 V; CE_max_, maximum current efficiency; CIE, Commission International del' Eclairage coordinates at 5 V; EL, electroluminescence; EQE_max/500/1000_, maximum EQE and the values taken at 500 and 1000 cd m^−2^; FWHM, full width at half maximum of the EL spectrum at 5 V; PE_max_, maximum power efficiency; *R*_max_, maximum radiant power; *V*_on_, turn on voltage at 1 cd m^−2^.

^a^ *R*_UV_ = UV_400_ × *R*_max_.

Owing to the well‐matched energy level alignment, devices N1 and D1 exhibit low turn‐on voltages of 3.4 and 3.6 V, with the maximum luminances of 1015 and 2179 cd m^−2^, respectively. As shown in Supporting Information S1: Figure S8, the luminance‐current density plots of devices N1 and D1 exhibit a good linear relationship with correlation coefficients exceeding 0.99, illustrating that the contribution of the TTA mechanism to triplet exciton utilization can be ruled out.^[53]^ Without out‐coupling enhancement or encapsulation, the non‐doped device N1 achieved maximum forward‐viewing efficiencies of 7.33% for EQE, 0.55 cd A^−1^ for current efficiency, and 0.51 lm W^−1^ for power efficiency at a luminance of 45.96 cd m^−2^. Remarkably, the EQE value of device N1 ranks among the highest reported for non‐doped UV OLEDs emitting below 400 nm (Figure 5f and Supporting Information S1: Table S4), which can be attributed to a high horizontal dipole orientation (84.0%) in the neat film of D3CZ4F (Supporting Information S1: Figure S9). Device D1 achieves a higher EQE of 8.97% at 323.50 cd m^−2^. The EQE remained at 8.15% and 6.63% at a high luminance of 500 and 1000 cd m^−2^, respectively, exhibiting a small roll‐off. Such small roll‐off can be contributed to the high oscillator strength of D3CZ4F, faster radiative transition process in the doped film and suppressed exciton quenching in the host‐guest system. Device N1 delivers stable UV emission with a peak at 390 nm and a narrow FWHM of 43 nm at 5 V, corresponding to CIE coordinates of (0.164, 0.027). The device D1 emits a blue‐shift UV light peaking at 388 nm with an FWHM of 42 nm and excellent color coordinates of (0.165, 0.025) (Supporting Information S1: Figure S10). To further evaluate the purity of UV‐OLEDs, here the parameter (UV_400_) is defined as the area proportion of the UV emission (*λ* ≤ 400 nm) in the EL spectrum. The device N1 and D1 exhibited high UV_400_ values of 59% and 64%, manifesting a high UV purity. The maximum radiant power (*R*_max_) values can be evaluated to be 58.47 mW cm^−2^ for device N1, 150.81 mW cm^−2^ for device D1, corresponding to the radiant power values from UV emission (*R*_UV_s) of 34.50 and 96.52 mW cm^−2^, respectively. It is worth noting that the high EQE of 8.15% at 500 cd m^−2^ achieved in the device D1 represents one of the highest efficiencies among reported UV‐OLEDs with EL peaks below 390 nm (Supporting Information S1: Table S5), making UV‐OLEDs potentially suitable for high brightness applications. However, owing to the current experimental constraints, the operational stability of the devices was not systematically evaluated in this work. For UV‐OLEDs, the device lifetime is critically compromised by a multitude of factors, including high‐energy exciton‐annihilation induced bond cleavage, Joule heating, morphological instability, material purity, device architecture, as well as fabrication and encapsulation protocols. Achieving commercially viable UV‐OLEDs ultimately demands a unified effort spanning fundamental research and industrial development.

## 3 CONCLUSION

In summary, we have developed a structurally simplistic yet functionally superior D–A–D type UV emitter, D3CZ4F, through a one‐step coupling reaction. The molecule integrates an HLCT excited state with multiple intramolecular non‐covalent C–H⋯F locking interactions, which endow D3CZ4F with a remarkable oscillator strength of 1.3989 and an exceptional PLQY of 98% while effectively enabling triplet exciton utilization and UV emission. The non‐doped OLED achieved a maximum EQE of 7.33% with an EL peak at 390 nm and CIE coordinates of (0.164, 0.027). When doped in a CBP host, D3CZ4F maintained a high PLQY of 86%, and the corresponding device delivered a peak EQE of 8.97% at 388 nm, exhibiting minimal efficiency roll‐off (EQE of 8.15% at 500 cd m^−2^). This work confirms that the introduction of a non‐covalent locking strategy into UV molecular design can effectively enhance the photoelectric performance, which provides a molecular design paradigm for the development of high‐efficiency and low‐cost UV emitters and OLEDs.

## 4 EXPERIMENTAL METHODS

### 4.1 Theoretical calculation methods

All the DFT and TD‐DFT calculations were carried out using Gaussian 16 A.03 package. The optimized ground‐state geometries, energy levels, and FMO distributions were calculated on the basis of DFT by using the B3LYP/6‐31G (d, p) method. The geometry of the *S*_1_ state was optimized by TD‐DFT at the B3LYP/6‐31G (d, p) level. Multifwn 3.8 was utilized to analyze the NTOs of excited states. SOC matrix elements were calculated by TD‐DFT and the ORCA 4.1.1 package at B3LYP/6‐31G (d, p).

### 4.2 Device fabrication and measurement

Patterned ITO glasses with a sheet resistance of 20 Ω per square were ultrasonically treated in detergents and deionized water and then dried for 30 mins at 120°C. After being treated by oxygen plasma for 7 mins, Clean ITO substrates were transferred into the vacuum deposition system. The target compounds used for the device have been sublimated. When the pressure was < 2 × 10^−4^ Pa, the devices were fabricated. The evaporation rates of organic materials LiF and Al were 1–1.5, 0.2 and 5–10 Å s^−1^ via a shadow mask, which were detected by a frequency counter and calibrated by a Dektak 6 M profiler (Veeco). The emitting area (3 × 3 mm^2^) was determined by the overlap between the ITO and Al electrodes. The current density–luminance–voltage characteristics were performed by a computer controlled Keithley 2450 series digital source‐meter and LS160 luminancemeter. EL spectra at different voltages were recorded by the optical analyzer FlAME‐S‐VIS‐NIR photometer. Supposing that the light emitted by the devices is accorded with the Lambertian distribution, the EQEs can be calculated from the EL spectra, luminance, and current density.

## CONFLICT OF INTEREST STATEMENT

The authors declare no conflicts of interest.

## ETHICS STATEMENT

No animal or human experiments were involved in this study.

## Supporting information

Supporting Information S1

## Data Availability

The data that support the findings of this study are available from the corresponding author upon reasonable request.
