# Supplementary material for: Facile one‐step synthesis of efficient hybridized local and charge transfer emitter for ultraviolet electroluminescence with low roll‐off
Source: Smart Mol. 2026 Aug 22:e70090. Online ahead of print. doi: 10.1002/smo2.70090 (PMC13499000; doi:10.1002/smo2.70090)
Supplement: Supplementary file 1 [file SMO2-9999-0-s001.docx]

Supporting Information

Facile one-step synthesis of efficient hybridized local and charge transfer emitter for ultraviolet electroluminescence with low roll-off

*Shengnan Wang, Jixiang Wang, Danyu Xie, Ling Peng,* Yuchao Liu, Junjie Wang, Shian Ying,* Dongge Ma, and Shouke Yan*

Shengnan Wang, Jixiang Wang, Yuchao Liu, Junjie Wang, Shian Ying, Shouke Yan

Key Laboratory of Rubber-Plastics, Ministry of Education, State Key Laboratory of Advanced Optical Polymer and Manufacturing Technology, Qingdao University of Science and Technology, Qingdao 266042, China

E-mail: shian0610@126.com; ORCID: 0000-0003-0781-240X

Danyu Xie, Dongge Ma

State Key Laboratory of Luminescent Materials and Devices, Guangdong Provincial Key Laboratory of Luminescence from Molecular Aggregates, Center for Aggregation-Induced Emission, South China University of Technology, Guangzhou 510640, China.

Ling Peng

College of Chemistry and Chemical Engineering, Heze University, Heze 274015, China.

E-mail: pengling615@163.com; ORCID: 0000-0003-4313-2954

Shouke Yan

State Key Laboratory of Chemical Resource Engineering, College of Materials Science and Engineering, Beijing University of Chemical Technology, Beijing 100029, P. R. China.

**S1. Materials and experimental procedures**

After acquisition, the raw materials and reagents were utilized without any additional purification. The target compound were finally purified by temperature-gradient sublimation (BOF-5-50, Anhui BEQ Equipment Technology Co, Ltd). Nuclear magnetic resonance (NMR) spectra, specifically ^1^H and ^13^C, were acquired using a Bruker AVAN CE NEO 400 spectrometer using tetramethyl silane (TMS) as the internal standard in chloroform*-d* (CDCl_3_). High resolution mass spectrometry (MS) measurements of the target materials were conducted using a quadrupole time-of-flight tandem mass spectrometer operating in MALDI-TOF mode. UV-visible (UV-vis) absorption spectra in dilute solutions were analyzed with a HITACHI U-4100 spectrophotometer. Photoluminescence (PL) spectra were recorded on an F-4700 fluorescence spectrofluorometer, while fluorescence lifetimes were measured using an Edinburgh FLS1000 transient fluorescence spectrometer. The PL quantum yields (PLQYs) of the target compound were determined using an integration sphere setup (Hamamatsu C11347-11) powered by a xenon high-pressure lamp. The angular-dependent spectra were measured with an ARM angle-resolved spectroscopy system (Idea Optics Instruments). Thermogravimetric analysis (TGA) was carried out using a Hitachi 7300 analyzer under a nitrogen atmosphere (50 mL min^-1^) at a heating rate of 20 °C min^-1^. Differential scanning calorimetry (DSC) curve was recorded by a Netzsch DSC (204F1) instrument at a heating (or cooling) rate of 10 °C min^‒1^. Cyclic voltammetry (CV) measurement was performed on a CHI660E electrochemical workstation in a standard three-electrode system comprising a glass carbon working electrode, a platinum wire auxiliary electrode, and an Ag/Ag^+^ reference electrode. Tetrabutylammonium hexafluorophosphate (Bu_4_NPF_6_, 0.1 M) in anhydrous dichloromethane (DCM) served as the supporting electrolyte, with ferrocene as the standard reference. The highest occupied molecular orbital (*E*_HOMO_) and lowest unoccupied molecular orbital (*E*_LUMO_) energy levels were calculated using the following formulas:

*E*_HOMO_ = −(*E*_ox_ – *E*_1/2_^+^ + 4.80) eV	(S1)

*E*_LUMO_ = (*E*_HOMO_ + *E*_g_) eV	(S2)

Here, *E*_ox_ represents the oxidation onset potential, *E*_1/2_^+^ denotes the half-wave potential of Fc/Fc^+^, and *E*_g_ is the optical bandgap derived from the absorption spectrum onset.

*Device fabrication and measurement:* The patterned indium-tin-oxide (ITO) glasses underwent ultrasonic cleaning in detergent and deionized water, followed by drying at 120 °C for 30 minutes. The cleaned ITO-substrates were then treated with oxygen plasma and transferred to the vacuum deposition system. Device fabrication took place in a high-vacuum chamber with a pressure maintained below 2 × 10^-4^ Pa. Evaporation rates were meticulously monitored using a frequency counter and calibrated with a Dektak 6 M profiler from Veeco. Organic materials, lithium fluoride (LiF), and aluminum (Al) were thermally evaporated through a shadow mask at controlled rates of 1-1.5 Å s^-1^, 0.2 Å s^-1^, and 5-10 Å s^-1^, respectively. The emitting area of the devices, defined by the overlap of the ITO and Al electrodes, was 3 × 3 mm^2^. To evaluate device performance, we measured current density, luminance, and voltage characteristics using a Keithley 2450 source meter and an LS160 luminance meter. Furthermore, electroluminescence (EL) spectra were captured using the FlAME-S-VIS-NIR photometer. Assuming Lambertian light emission from the OLEDs, we estimated the external quantum efficiencies (EQEs) based on the EL spectra, luminance, and current density measurements.

*Theoretical calculation methods:* All density functional theory (DFT) and time-dependent DFT (TD-DFT) computations for the target materials were performed using the Gaussian 09 software. Within the DFT framework, the B3LYP/6-31G(d, p) basis set was employed to determine the optimized ground-state geometries, energy levels, and frontier molecular orbital (FMO) distributions. Additionally, TD-DFT calculations using the same B3LYP/6-31G(d, p) method were conducted to obtain the optimized excited-state geometries, natural transition orbitals (NTOs), energies of singlet and triplet states, and oscillator strengths. Spin-orbit coupling (SOC) matrix elements were calculated using TD-DFT in the ORCA 4.1.1 package with the B3LYP/6-31G(d, p) method. The Visual Molecular Dynamics (VMD) program was utilized for graphics and visualization analyses. Furthermore, the contributions of charge transfer (CT) to each excited state were evaluated by calculating the charges of donor and acceptor fragments using the Hirshfeld partition model, corrected for interfragment charge transfer (IFCT), with the assistance of the Multiwfn program. The reorganization energies for the ground (S_0_) and first singlet excited (S_1_) states were decomposed based on geometry optimizations performed using density functional theory. The coupling between structural relaxation and vibrational modes was quantified in terms of Huang–Rhys (HR) factors, which are calculated as follows:

$S_{i}=\frac{\lambda_{i}}{{h\omega}_{i}}$	(S3)

where $S_{i}$ is the Huang–Rhys factor for the $i$-th vibrational mode, $\lambda_{i}$ denotes the corresponding contribution to the total reorganization energy, and $\omega_{i}$ represents the vibrational frequency of that mode.

**S2. Synthesis route and characteristic**

***Synthesis of 3,3'-(perfluoro-1,4-phenylene)bis(9-phenyl-9H-carbazole) (******D3CZ4F)***

Under a nitrogen atmosphere 1,4-dibromo-2,3,5,6-tetrafluorobenzene (1.54 g, 5.01 mmol), (9-phenyl-9*H*-carbazol-3-yl)boronic acid (3.02 g, 10.52 mmol), and the catalyst tetrakis(triphenylphosphine)palladium (0.23 g, 0.20 mmol) were dissolved in a mixture of 30 mL toluene, 10 mL ethanol, and 10 mL potassium carbonate solution. The reaction mixture was heated to 110 °C and stirred for 24 hours. After extracted with dichloromethane and water three times. The collected organic layer was concentrated using a rotary evaporator. The resulting residue was purified by column chromatography on silica gel, employing a DCM/ petroleum ether (PE) mixture (1/2, v/v) as the eluent. By further purified using temperature-gradient sublimation, yielding a white solid powder (2.21 g, yield: 69.8%). ^1^H NMR (400 MHz, CDCl_3_) δ 8.34 (s, 2H), 8.20 (d, *J* = 7.8 Hz, 2H), 7.59 (dt, *J* = 29.7, 9.2 Hz, 14H), 7.45 (d, *J* = 5.7 Hz, 4H), 7.35 (d, *J* = 7.6 Hz, 2H). ^13^C NMR (101 MHz, CDCl_3_) δ 141.38, 140.98, 137.37, 130.02, 127.92, 127.82, 127.18, 126.48, 123.58, 123.14, 122.43, 120.50, 120.41, 118.98, 110.05, 109.96. MALDI-TOF-MS(m/z): [M]^+^ calcd for C_42_H_24_F_4_N_2_: 632.1876; Found: 632.1880.

**Scheme S1.** Synthesis route of target material D3CZ4F.

**S3. Thermal and electrochemical analysis**

**Figure S1.** (a) DSC curve of D3CZ4F. (b) TGA curve of D3CZ4F.

**Figure S2.** CV curve of D3CZ4F.

**S4. Quantum chemical calculation**

**Table S1.** Energy levels, proportion of CT component and transition configuration of singlet and triplet states for D3CZ4F in a vacuum. And the oscillator strengths of singlet states.

| **Excited States** | **S energy level**  **[eV]** | **CTs**  **[%]** | **S Oscillator Strength** | **Transition configuration of S** | **T energy level**  **[eV]** | **CTs**  **[%]** | **Transition configuration of T** |
| --- | --- | --- | --- | --- | --- | --- | --- |
| 1 | 3.666 | 34.03 | 1.3989 | H → L 97.1% | 2.890 | 19.37 | H → L 60.9%  H-5 → L 9.4%  H-2 → L+2 6.1%  H-3 → L+1 5.5% |
| 2 | 3.898 | 34.40 | 0.0459 | H → L+1 64.6%  H-1 → L+2 17.2%  H-3 → L 11.2% | 3.153 | 14.15 | H-3 → L+2 29.0%  H-2 → L+1 26.0%  H-1 → L 13.2%  H → L+7 9.2% |
| 3 | 3.903 | 32.05 | 0.0000 | H → L+2 62.5%  H-1 → L+1 16.5%  H-2 → L 13.0% | 3.264 | 23.73 | H-3 → L+1 22.8%  H → L+2 22.3%  H-2 → L+2 14.4%  H-1 → L+1 8.8%  H → L 6.6% |
| 4 | 3.933 | 45.09 | 0.0000 | H-1 → L 92.0% | 3.312 | 28.43 | H → L+1 50.9%  H-1 → L+2 27.6%  H-1 → L 6.4% |
| 5 | 4.237 | 52.45 | 0.0000 | H → L+3 71.9%  H-1 → L+4 20.3% | 3.384 | 27.04 | H → L+2 32.9%  H-1 → L+1 27.4%  H-2 → L+2 13.5%  H-3 → L+1 8.5% |
| 6 | 4.239 | 40.96 | 0.1162 | H-2 → L 73.1%  H → L+2 6.1%  H-1 → L+1 5.9% | 3.494 | 28.72 | H → L+3 21.1%  H-1 → L 15.7%  H-1 → L+4 12.1%  H-1 → L+2 6.9%  H-8 → L+4 6.4%  H-7 → L+3 6.3% |
| 7 | 4.269 | 49.32 | 0.0000 | H → L+4 65.7%  H-1 → L+3 26.0% | 3.548 | 22.93 | H → L+4 23.0%  H-1 → L+3 22.0%  H-8 → L+3 11.0%  H-7 → L+4 8.9%  H-9 → L+5 5.2%  H-10 → L+6 5.0% |
| 8 | 4.300 | 69.71 | 0.0080 | H → L+5 68.5%  H-1 → L+5 26.8% | 3.686 | 34.99 | H-1 → L 32.0%  H-2 → L+1 10.0%  H-1 → L+4 9.5%  H-3 → L+2 7.2%  H → L+7 6.1% |
| 9 | 4.301 | 67.46 | 0.1500 | H → L+6 69.4%  H-1 → L+6 26.1% | 3.829 | 35.77 | H-2 → L 49.5%  H-2 → L+2 8.7%  H → L 8.2%  H-1 → L+7 5.7% |
| 10 | 4.320 | 48.36 | 0.0001 | H-3 → L 58.5%  H → L+1 24.6%  H-1 → L+2 10.6% | 3.860 | 33.63 | H-3 → L 62.0%  H-2 → L+7 7.1% |

**S5. Photophysical properties**

**Figure S3.** The steady-state PL spectra of D3CZ4F in chlorobenzene (10^−4^ M) in N_2_ and air atmospheres at 300 K.

**Figure S4.** Absorption spectra of D3CZ4F in different solvents (1 × 10^-5^ M).

**Figure** **S5.** Transient PL decay spectra of D3CZ4F-based neat film and doped film in the window of 50 μs.

**Table S2.** The detailed absorption and emission peaks of D3CZ4F in different solvents.

| **Solvents** | **ε** | **n** | ***f* (ε, n)** | **D3CZ4F** | | |
| --- | --- | --- | --- | --- | --- | --- |
|  |  |  |  | **λ_A_ ^a)^ (nm)** | **λ*_PL_* ^b)^ (nm)** | **ν_A_- ν*_PL_* ^c)^ (cm^-1^)** |
| Hexane | 1.9 | 1.375 | 0.0012 | 294 | 367 | 6765.65 |
| Toluene | 2.38 | 1.494 | 0.013 | 296 | 380 | 7467.99 |
| Triethylamine | 2.42 | 1.401 | 0.048 | 296 | 372 | 6902.06 |
| Butyl ether | 3.08 | 1.399 | 0.096 | 294 | 375 | 7346.94 |
| Isopropyl ether | 3.88 | 1.368 | 0.145 | 294 | 381 | 7766.89 |
| Ethyl ether | 4.34 | 1.352 | 0.167 | 294 | 376 | 7417.86 |
| Ethyl acetate | 6.02 | 1.372 | 0.2 | 294 | 385 | 8039.58 |
| Tetrahydrofuran | 7.58 | 1.407 | 0.21 | 294 | 388 | 8240.41 |
| Dichloromethane | 8.93 | 1.424 | 0.217 | 294 | 389 | 8306.66 |
| Dimethylformamide | 37 | 1.427 | 0.276 | 296 | 402 | 8908.16 |
| Acetone | 20.7 | 1.359 | 0.284 | - | 396 | - |
| Acetonitrile | 37.5 | 1.344 | 0.305 | 292 | 398 | 9120.95 |

^a)^ The absorption peak measured in dilute different solutions (1.0 × 10^−5^ M) at 300 K. ^b)^ The photoemission peak measured in dilute different solutions (1.0 × 10^−5^ M) at 300 K. ^c)^ (ν_A_- ν*_PL_*) = (1/λ_A_) − (1/λ*_PL_*).

**Table S3.** Kinetic parameters of D3CZ4F in neat and doped films.

| **Film** | ***Φ_PL_* ^a)^**  **[%]** | **τ_PF_ ^b)^**  **[ns]** | **τ_DF_ ^c)^**  **[ns]** | ***k*_r_ ^d)^**  **[×10^8^ s^-1^]** | ***k*_nr_ ^e)^**  **[×10^8^ s^-1^]** | ***k*_ISC_ ^f)^**  **[×10^8^ s^-1^]** | ***k*_hRISC_ ^g)^**  **[×10^8^ s^-1^]** |
| --- | --- | --- | --- | --- | --- | --- | --- |
| **Neat film** | 25 | 1.10 | 4.48 | 1.85 | 5.55 | 1.69 | 0.27 |
| **Doped film** | 86 | 1.07 | 5.23 | 6.96 | 1.13 | 1.25 | 0.22 |

^a)^ Photoluminescence quantum yield (*Φ_PL_*). ^b)^ Prompt fluorescence lifetime. ^c)^ Delayed fluorescence lifetime. ^d)^ Rate of radiative transition (*k*_r_ = *Φ*_PF_/*τ*_PF_). ^e)^ Rate of non-radiative transition (*k*_nr_ = *k*_r_/*Φ_PL_* − *k*_r_). ^f)^ Rate of intersystem crossing (*k_ISC_ = k*_r_*/Φ*_PF_ − *k*_r_ − *k*_nr_). ^g)^ Rate of reverse intersystem crossing (*k*_RISC_ =(*k*_PF_*k*_DF_*Φ*_DF_)/(*k*_ISC_*Φ*_PF_)).

**S6. OLED device and electroluminescent performance**

**Figure S6.** Chemical structure of organic materials used in the OLEDs.

**Figure S7.** Power efficiency-luminance-current efficiency characteristics of devices N1 and D1.

**Figure S8.** Luminance‒current density characteristics and their linear fits (R^2^ > 0.99) for devices N1 and D1.

**Figure S9.** (a) Experimentally measured angle-dependent PL spectra of D3CZ4F-based neat film (a) and doped film (b), and simulated curves with 100% and 67% dipole orientation ratios (Θ).

**Figure S10.** (a) CIE coordinates of N1 at 5 V. (b) CIE coordinates of D1 at 5 V.

**Table S4.** Summary of reported non-doped UV-OLEDs with an EL peak ≤ 400 nm in recent years.

| **Compounds** | **EQE_max_ [%]** | **EL peak [nm]** | **FWHM**  **[nm]** | **CIE_y_** | **Ref.** |
| --- | --- | --- | --- | --- | --- |
| **D3CZ4F** | **7.33** | **390** | **43** | **0.027** | **This work** |
| 2Na-CzCN | 5.92 | 398 | 53 | 0.06 | [1] |
| mP2MPC | 6.09 | 376, 395 | 51 | 0.028 | [2] |
| 4DBF-CZCN | 3 | 390 | 42 | 0.034 | [3] |
| 2DBF-CZCN | 2.86 | 388 | 38 | 0.036 | [3] |
| DCz-mPy-Na | 4 | 388 | 43 | 0.048 | [4] |
| DCz-pPy-Na | 2.21 | 393 | 53 | 0.065 | [4] |
| DCz-mPy-PhCz | 4.07 | 395 | 39 | 0.044 | [4] |
| cis-trans-TWG1 | 4.17 | 370 | 34 | 0.047 | [5] |
| mPIoCZ2F | 6.62 | 393 | 48 | 0.034 | [6] |
| DBC8 | 3.75 | 376 | 32 | 0.029 | [7] |
| YJCO | 3.34 | 380 | - | 0.04 | [7] |
| YJNH | 3.46 | 385 | - | 0.04 | [7] |
| BuICz | 0.99 | 390 | 36.7 | 0.043 | [8] |
| BB4Ph | 5.24 | 386, 400 | - | 0.045 | [9] |
| 2,7PC9D | 6.53 | 389 | 42 | 0.028 | [10] |
| 2,7PC9N | 4.81 | 383 | 40 | 0.026 | [10] |

**Table S5.** Summary of reported UV-OLEDs with an EL peak ≤ 390 nm in recent years.

| **Compound** | **FWHM**  **[nm]** | **EL peak**  **[nm]** | **EQE_max_**  **[%]** | **EQE_500_**  **[%]** | **CIE_y_** | **Ref.** |
| --- | --- | --- | --- | --- | --- | --- |
| **D3CZ4F** | **42** | **388** | **8.97** | **8.15** | **0.025** | **This work** |
| BFCz-pPy | 43 | 388 | 6.4 | 6.3 | 0.032 | [11] |
| DMID-mPy | 39 | 387 | 7.88 | 7.66 | 0.031 | [11] |
| BFCz-mPy | 41 | 384 | 7.9 | 7.59 | 0.029 | [11] |
| DCz-*m*Py-S | 40 | 378 | 7.15 | 6.96 | 0.029 | [12] |
| mCz | 38 | 382 | 10.6 | <5% | 0.042 | [13] |
| TAZ | 37 | 376 | 4.14 | <5% | - | [14] |
| DSiPPI | - | 377 | 7.07 | <5% | 0.078 | [15] |
| ICZ-TAZ | 32 | 388 | 3.3 | <5% | 0.069 | [16] |
| 3,6PC9D | 36 | 379 | 7.18 | 5.99 | 0.028 | [10] |
| 3,6PC9N | 36 | 384 | 7.39 | 6.29 | 0.029 | [10] |
| BuICz | 33.3 | 390 | 2.76 | <5% | 0.084 | [8] |
| BuICzCN | 32.3 | 388 | 3.92 | <5% | 0.093 | [8] |
| mCzPI | 38 | 376/395 | 3.78 | <5% | 0.028 | [17] |
| mMCzPI | 38 | 376/393 | 3.03 | <5% | 0.027 | [17] |
| oCzPI | 56 | 388 | 3.04 | <5% | 0.045 | [17] |

**S7. ^1^H NMR spectrum**

**Figure S11.** The ^1^H NMR spectrum of D3CZ4F in CDCl_3_.

**S8. References**

[1] G. Li, B. Li, H. Zhang, X. Guo, C. Lin, K. Chen, Z. Wang, D. Ma, B.Z. Tang, *ACS Appl. Mater. Interfaces* 2022, **14**, 10627-10636.

[2] L. Peng, J.C. Lv, S. Xiao, Y. Huo, Y. Liu, D. Ma, S. Ying, S. Yan, *Chem. Eng. J* 2022, **450**, 138339.

[3] H. Huang, R. Chen, S.Y. He, S. Wang, H. Qi, L. Peng, Y. Liu, S. Ying, S. Yan, *Dyes. Pigm.* 2024, **231**, 112420.

[4] Y.M. Huo, L. Peng, S.Y. He, R. Chen, H. Huang, F. He, Y. Ming, Y. Liu, S. Ying, S. Yan, *Dyes. Pigm.* 2024, **226**, 112128.

[5] Y. Wei, C.X. Zhong, Y. Sun, S. Ma, M. Ni, X. Wu, Y. Yan, L. Yang, I.A. Khodov, J. Ge, Y. Li, D. Lin, Y. Wang, Q. Bao, H. Zhang, S. Wang, J. Song, J. Lin, L. Xie, W. Huang, *Nat. Commun.* 2024, **15**, 5438.

[6] S.N. Wang, R. Zhang, R.J. Ding, H. Huang, H. Qi, Y. Liu, S. Ying, D. Ma, S. Yan, *Chem. Sci.* 2025, **16**, 5518-5527.

[7] J. Yang, J.L. Lou, M.J. Ni, Z. Zhuo, H. Li, X. An, N. Sun, M. Li, X. Ding, L.Wang, M. Xu, X. Ran, Z. Wang, J. Lin, W. Huang, *Chem. Eng. J* 2025, **524**, 168961.

[8] K.Y. Lu, J.W. Huang, X. Chang, K. Jiang, X. Yan, X. Wang, J. Yu, J. Zhu, X. Wu, W. Zhu, *Appl. Phys. Lett.* 2024, **124**, 151902.

[9] J.H. Lee, J.X. Huang, C.H. Chen, Y.T. Lee, C.Y. Chan, Y.C. Dzeng, P.W. Tang, C. Chen, C. Adachi, T.L. Chiu, J.H. Lee, C.T. Chen, *Adv. Opt. Mater.* 2023, **11**, 2202666.

[10] H. Huang, R. Zhang, R.J. Ding, D. Xie, Y. Liu, J. Wang, S. Ying, D. Ma, S. Yan, *Dyes. Pigm.* 2026, **246**, 113446.

[11] S.Y. He, R.J. Ding, S.N. Wang, L. Xia, Y. Liu, J. Wang, S. Ying, D. Ma, S. Yan, *ACS Appl. Mater. Interfaces* 2026, **18**, 8529–8538.

[12] S.Y. He, L. Peng, M.H. Zhao, S. Wang, Y. Liu, J. Wang, S. Ying, S. Yan, *Dyes. Pigm.* 2026, **251**, 113778.

[13] J.L. Lou, X. Guo, Y. Chen, H. Zhang, D. Yang, D. Ma, B.Z. Tang, Z. Wang, *Chem. Eng. J* 2025, **515**, 163817.

[14] F. You, B. Mo, L. Liu, H. Wang, B. Wei, J. Xu, X. Zhang, *Opt. Laser. Technol.* 2016, **82**, 199-202.

[15] Y. Zheng, X. Zhu, Z. Ni, X. Wang, Z. Zhong, X.J. Feng, Z. Zhao, H. Lu, *Adv. Opt. Mater.* 2021, **9**, 2100965.

[16] X. He, J. Lou, B. Li, H. Wang, X. Peng, G. Li, L. Liu, Y. Huang, N. Zheng, L. Xing, Y. Huo, D. Yang, D. Ma, Z. Zhao, Z. Wang, B.Z. Tang, *Angew. Chem. Int. Ed.* 2022, **61**, e202209425.

[17] Z. Zhong, Z. Liu, X. Wang, D. Xiong, H. Li, X.J. Feng, Z. Zhao, H. Lu, *J Mater. Chem. C* 2023, **11**, 16271-16279.
